# Charge-Dependent Regulation in DNA Adsorption on 2D Clay Minerals

**DOI:** 10.1038/s41598-019-41093-5

**Published:** 2019-05-02

**Authors:** Hongyi Xie, Zhengqing Wan, Song Liu, Yi Zhang, Jieqiong Tan, Huaming Yang

**Affiliations:** 1 Department of Inorganic Materials, School of Minerals Processing and Bioengineering, Central South University, Changsha, 410083 China; 2 Hunan Key Lab of Mineral Materials and Application, Central South University, Changsha, 410083 China; 3 The Center for Medical Genetics, School of Life Science, Central South University, Changsha, 410078 China; 4 Institute of Chemical Biology and Nanomedicine, College of Chemistry and Chemical Engineering, Hunan University, Changsha, 410082 China

**Keywords:** Biomaterials, Structural materials

## Abstract

DNA purification is essential for the detection of human clinical specimens. A non-destructive, controllable, and low reagent consuming DNA extraction method is described. Negatively charged DNA is absorbed onto a negatively charged montmorillonite to achieve non-destructive DNA extraction based on cation bridge construction and electric double layer formation. Different valence cation modified montmorillonite forms were used to validate the charge-dependent nature of DNA adsorption on montmorillonite. Electric double layer thickness thinning/thickening with the high/lower valence cations exists, and the minerals tended to be sedimentation-stable due to the Van der Waals attraction/electrostatic repulsion. Li-modified montmorillonite with the lowest charge states showed the best DNA adsorption efficiency of 8–10 ng/μg. Charge-dependent regulating research provides a new perspective for controllable DNA extraction and a deep analysis of interface engineering mechanisms.

## Introduction

DNA purification has been widely used for transfection, sequencing, and polymerase chain reaction (PCR)^1^. Sample preparation steps, including adsorption and desorption of DNA are of prime importance for the following analysis procedures, using the physicochemical characteristics of the negative charge (owing to the phosphate group (PO_4_^3−^))^2,3^ and the different solubilities in organic and aqueous phases. Traditional chemical extraction, magnetic separation^4–9^ and silicon material matrix separation, such as SiO_2_^10–15^ and other extraction methods^16–19^ (the corresponded adsorption pattern is shown in Table S1), have been developed over the past decades. Generally, surface charging is the principal method currently available for strengthening the interaction between DNA and materials in the solid phase extraction process. Despite rapid advances in performance improvement of DNA extraction efficiency, less attention has been paid to the influence of the properties of modified materials at the same time. Therefore, a conceptually different method was employed for charge-dependent extraction of DNA based on the DLVO theory (a dispersion stabilizing theory).

Natural clay, as an emerging biomaterial for biomedical engineering^20–29^, has the advantages of stable chemical composition^30,31^, various morphology structures and the specific physicochemical characteristics^32–34^. Montmorillonite (MMT, Al_2_Si_4_O_10_(OH)_4_·nH_2_O) is a 2:1 layered clay mineral that consists of one Al-octahedral sheet sandwiched between two Si-tetrahedral sheets^31,35^. The natural negative charge is derived from the crystalline substitution, and the charge state could be altered by electric double layer regulation or cation exchange. In addition, the broken edges of silicate clay minerals adsorb hydrogen ions in different pH medium environments and could affect the charge. The unique structure and natural characteristics^36,37^ expand its applied range for biomedical engineering^38–41^.

**Figure 1:**
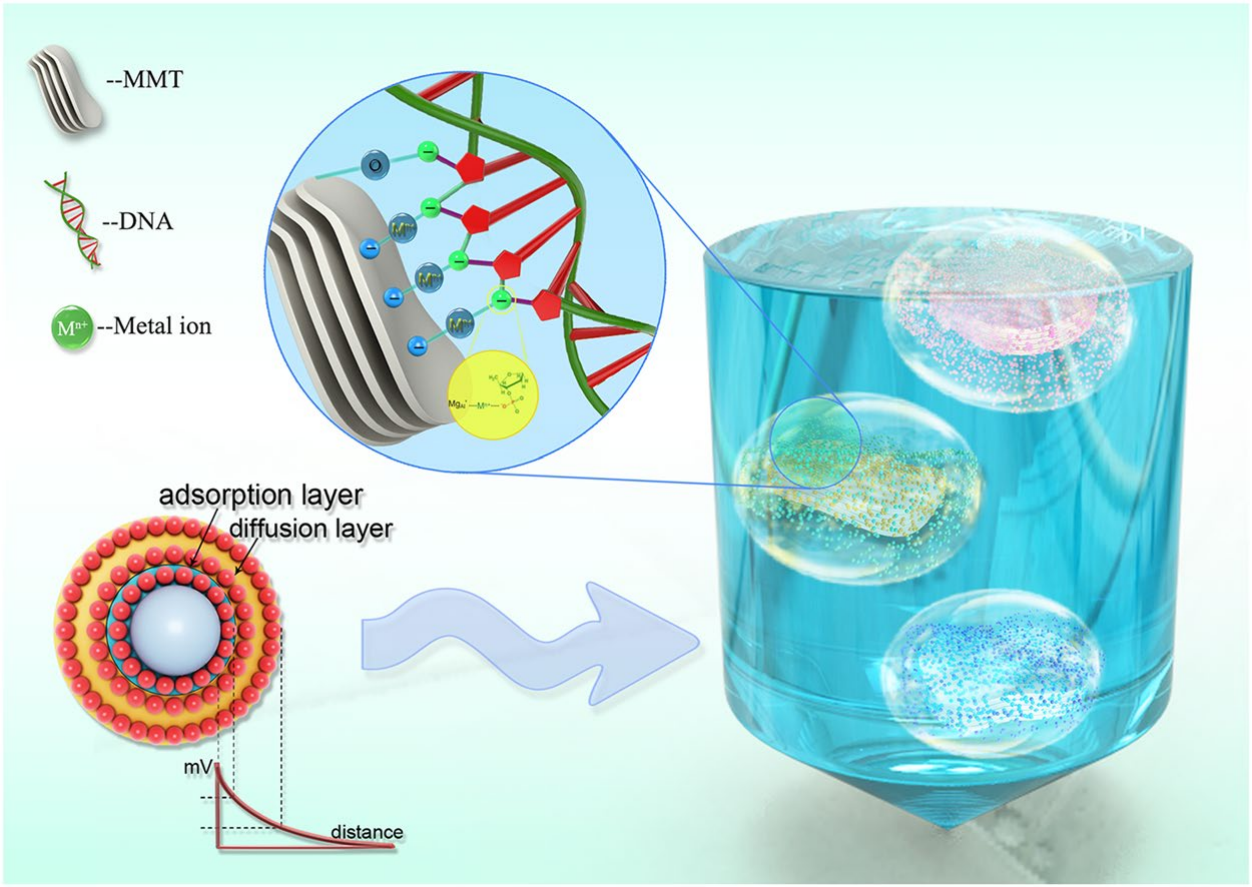
Schematic representation of fabrication of ion-MMT and the adsorption of DNA.

Obviously, the positive charge facilitates adsorption by the strong electrostatic attraction between the opposite electricity. However, it is unclear whether the negative electricity will serve as a regulator of the adsorption process. It is easy to see that the adsorption between two negative materials by weaker electrostatic attraction, such as a cation bridge, is more conducive to non-destructive desorption. Inspired by this idea, negatively charged DNA (purified from HeLa cells) is absorbed onto the negatively charged montmorillonite to achieve a non-destructive DNA extraction based on the cation bridge construction and the electric double layer formation (Fig. 1). An ideal single factor analysis is established. The adsorption was highly correlated with charge state. The relationship between the different valence cations and the electric double layer thickness formation under the DLVO theory was clarified. The charge-dependent and surface-interfacial relationship in DNA adsorption was detailed. Further, the relationships among the charge, the dispersion, and the cation bridge for MMT interactions with DNA were analysed.

## Results

**Figure 2:**
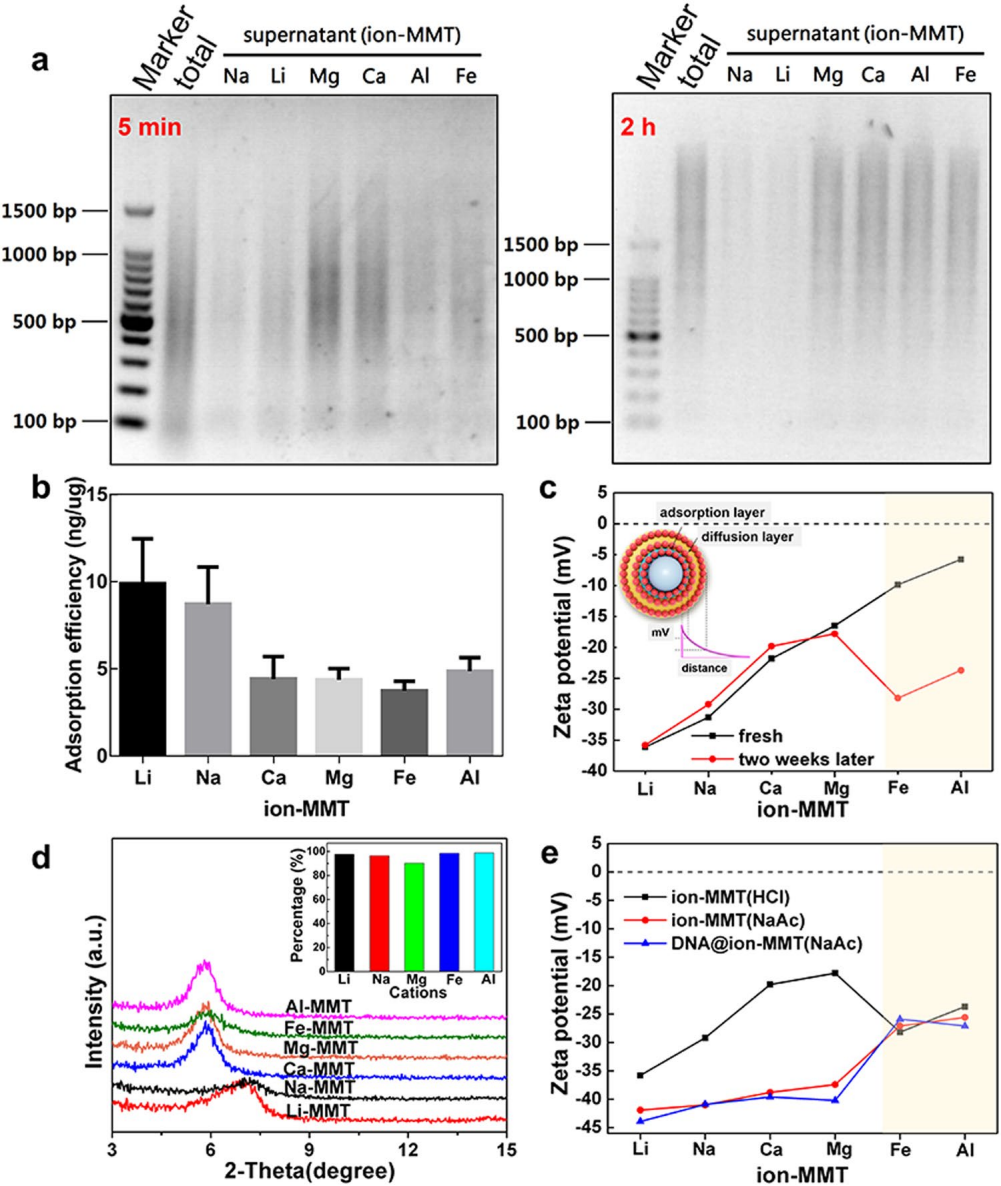
Adsorption and characterization of ion-MMT: (**a**) Electrophoresis analysis for the supernatant of clay binding with DNA (the intact gels of adsorption and desorption were included in a supplementary information file as shown in Fig. S1). (**b**) Adsorption efficiency of DNA of different samples. (**c**) Zeta potential of fresh samples and samples after a week. (**d**) XRD pattern of Na-MMT, Li-MMT, Ca-MMT, Mg-MMT, Fe-MMT and Al-MMT; the inset shows the modification efficiency of different cations measured by ICP. (**e**) Zeta potential of samples in different system and binding with DNA.

Different valence cations (Li^+^, Na^+^, Mg^2+^, Fe^3+^ and Al^3+^) with the same mass ratio were selected to modify the Ca-MMT to the maximum adsorption capacity, and the corresponding adsorption was measured. The adsorption and desorption of DNA could be achieved by different ion-MMT combinations (Fig. 2a and Fig. S1). Based on the unchanged brightness of agarose gel electrophoresis, the adsorption efficiency of Li-MMT, Na-MMT, Ca-MMT and Mg-MMT were not changed as time increased. Interestingly, the DNA adsorption capacity of Al-MMT and Fe-MMT varied from 5 min to 2 hours, due to the sedimentation of the Al or Fe ion hydrolysis, as indicated by the different brightness of two agarose gel electrophoresis bands. To further investigate the adsorption efficiency, Nano Drop software is utilized based on the brightness of the lines showed in Fig. 2a. Na-MMT and Li-MMT exhibit high DNA binding action with an 8–10 ng/μg adsorption capacity (Fig. 2b), and Mg-MMT and Ca-MMT show only a 4–5 ng/μg adsorption capacity.

To further explore the possible interfacial interactions between ion-MMT and DNA, zeta potential was obtained to mirror the particles’ surface charge, and a surprising observation was made. The zeta potential of Al-MMT and Fe-MMT was changeable when the ion-MMT was remeasured two weeks later. The results showed that the zeta potential value of ion-MMT increased gradually as the cationic valence increased due to the thinner diffusion layer formed by the high cationic valence state (Fig. 2c, black line). The high valence cations would compress the thickness of the electric double layer, thus improving the zeta potential. Interestingly, the zeta potential of Al-MMT and Fe-MMT were markedly decreased from −5.76 mV and −9.85 mV to −23.7 mV and −28.2 mV after two weeks, respectively, which could be attributed to the hydrolysis of Fe^3+^ and Al^3+^ (Fig. 2c).

To determine the ions locations during the modification process, ICP and XRD were used to evaluate the ion-exchanged efficiency of MMT. XRD analysis (Figs 2d and S2) revealed that the typical diffraction pattern of Ca-MMT with a characteristic d_001_ value of 1.49 nm (2θ = 5.92°), shifted to 1.30 nm (7.18°), 1.29 nm (6.84°), 1.44 nm (6.12°), and 1.52 nm (5.80°), which corresponds to Na-MMT, Li-MMT, Fe-MMT and Al-MMT, respectively. The above results indicate obvious shifting with successful intercalation, slight shifting with inadequate intercalation with the most adsorbed on the external surface, and a lack of shifting, it was mainly adsorbed on the external surface (Mg-MMT). The ICP results showed that 90% of the cations were successfully modified to MMT (inset in Fig. 2d).

In addition, we found that the zeta potential of the ion-MMT in a hydrochloric acid (black) and sodium acetate (red) solution system showed different behaviours, with the exception of Fe-MMT and Al-MMT (Fig. 2e), which could be attributed to the diffusion layer thickening when the cation ions captured the negatively charged acetate groups. However, the trivalent cation ions were limited by the ion hydrolysis, and the surface cations decreased. Thus, the diffusion layer could not thicken, and the zeta potential was generally not changed. In addition, the charge state of ion-MMT@DNA remains negative and even lower than ion-MMT. Interestingly, the adsorption capacity was greatest for the lowest charge states.

**Figure 3:**
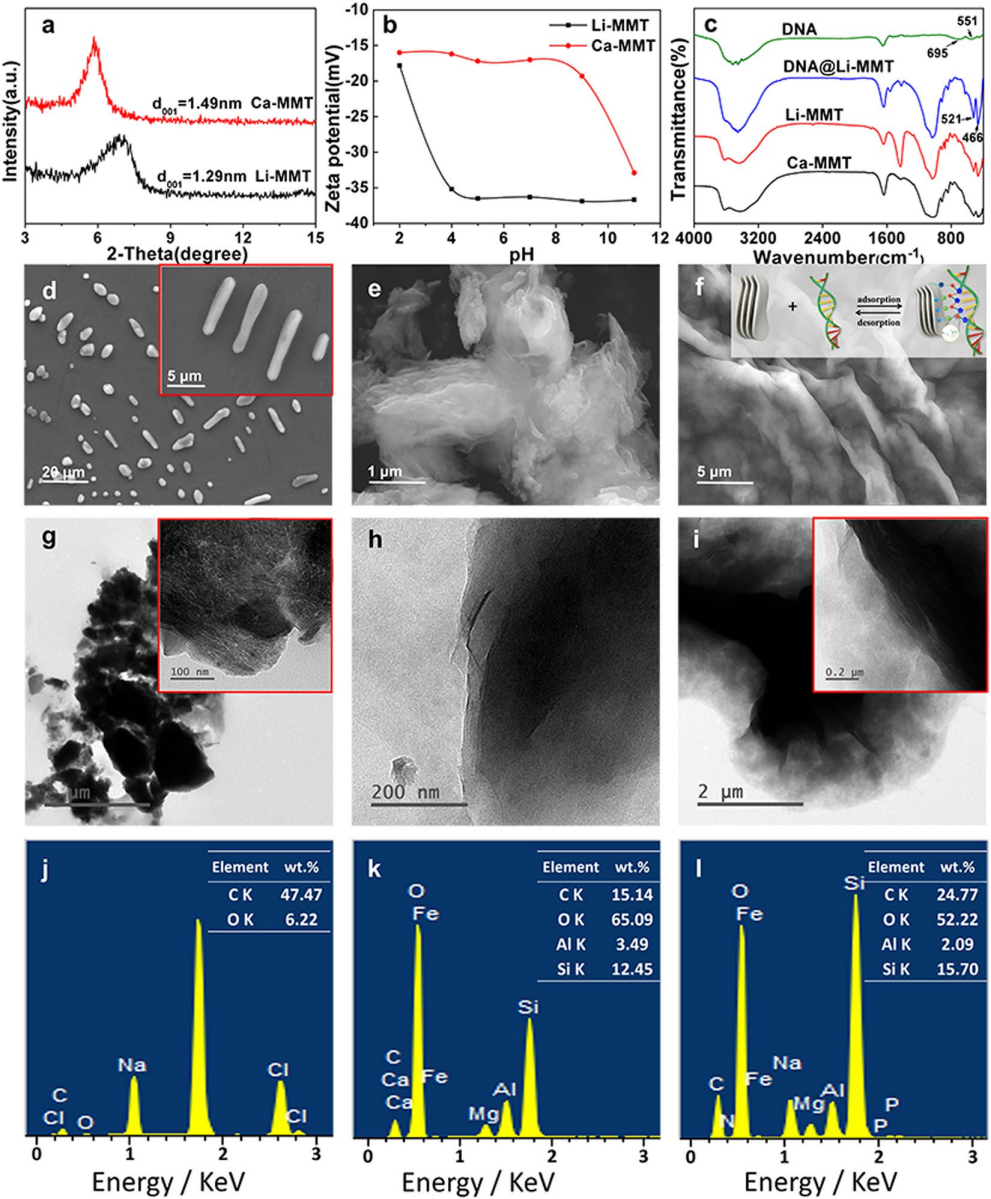
Characterization of Li-MMT before and after the adsorption of DNA: (**a**) XRD pattern and (**b**) Zeta potential of Li-MMT and Ca-MMT. (**c**) FTIR spectra of DNA, Ca-MMT, Li-MMT before and after binding with DNA. (**d**–**f**) SEM images of DNA, Li-MMT and Li-MMT@DNA; (**g**–**i**) TEM images of DNA, Li-MMT and Li-MMT@DNA; (**j**–**l**) is the SEM EDX confirming the formation of DNA, Li-MMT and Li-MMT@DNA.

The interlayer spacing of Li-MMT was decreased (Fig. 3a) and the zeta potential was negative and stable at pH >4 (Fig. 3b) when compared with the original d_001_ value = 1.49 nm, which was stable at approximately pH = 11 for Ca-MMT. The corresponding FTIR spectrums results (Fig. 3c and Fig. S3) showed that the O-H stretching vibration band at 3,620 cm^−1^ and 3,420 cm^−1^ correspond to the Al-OH group and the interlayer water molecule, respectively. Bending-vibration bands of O-H are observed at 1,636 cm^−1^ and 3,420 cm^−1^, which indicates that the ion-MMT interlayer contains crystal water. The stretching-vibration band of Si-O at 1,035 cm^−1^ and the bending-vibration band of Si-O at 465 cm^−1^ indicated that the layered structure of ion-MMT consisted of a silicon-oxygen tetrahedron. The bending-vibration bands of Al-OH, Mg-OH, Si-OMg and Si-OFe were located at 915, 844, 525 and 460 cm^−1^, respectively. For DNA obtained using phenol-chloroform extraction, the band at 551 cm^−1^ corresponded to the asymmetric variable angle of PO_4_, and the band at 695 cm^−1^ was ascribed to the stretching-vibration of P = S given the remaining protein during the extraction. The sharp bands at 3,510 and 3,455 cm^−1^ are ascribed to the anti-symmetrical and symmetrical stretching vibration of O-H, and the band at 1,649 cm^−1^ corresponds to the variable angle vibration of O-H for the tested DNA when dissolved in water. For the DNA@ion-MMT samples, the asymmetric variable angle of PO_4_ is shifted to 624 cm^−1^. The bending-vibration band of Si-OMg and Si-OFe were stronger and shifted to 521 and 466 cm^−1^ after combining with DNA. The other vibration band in the FTIR spectrum of DNA@ion-MMT was similar to that of ion-MMT.

SEM and TEM images with the EDX were used to survey the interfacial relationships between DNA and MMT, and the findings are presented in Fig. 3. DNA has the morphology of a short stick or elliptical with an average length of 1–20 μm, which is attributed to the agglomeration of single DNA (5–6 μm in length and 2 nm in diameter) (Fig. 3d). The TEM image of DNA indicates that the white-threadlike DNA was filamentous clumped together and formed an organic-like film (Fig. 3g). The energy-dispersive X-ray (EDX) spectra demonstrated that the DNA mainly contains C and O (Fig. 3j). Li-MMT has the morphology of a typical layered structure that was 3–4 μm in width and 1–2 μm in thickness (Fig. 3e,h). After the DNA was adsorbed, a more organic-like film was wrapped on the layered structure (Fig. 3f,i), with rare white-threadlike matters on the end edge of the Li-MMT, and the carbon content was increased from 15.14 wt.% to 24.77 wt.% due to the C element in the DNA (Fig. 3k,l).

**Figure 4:**
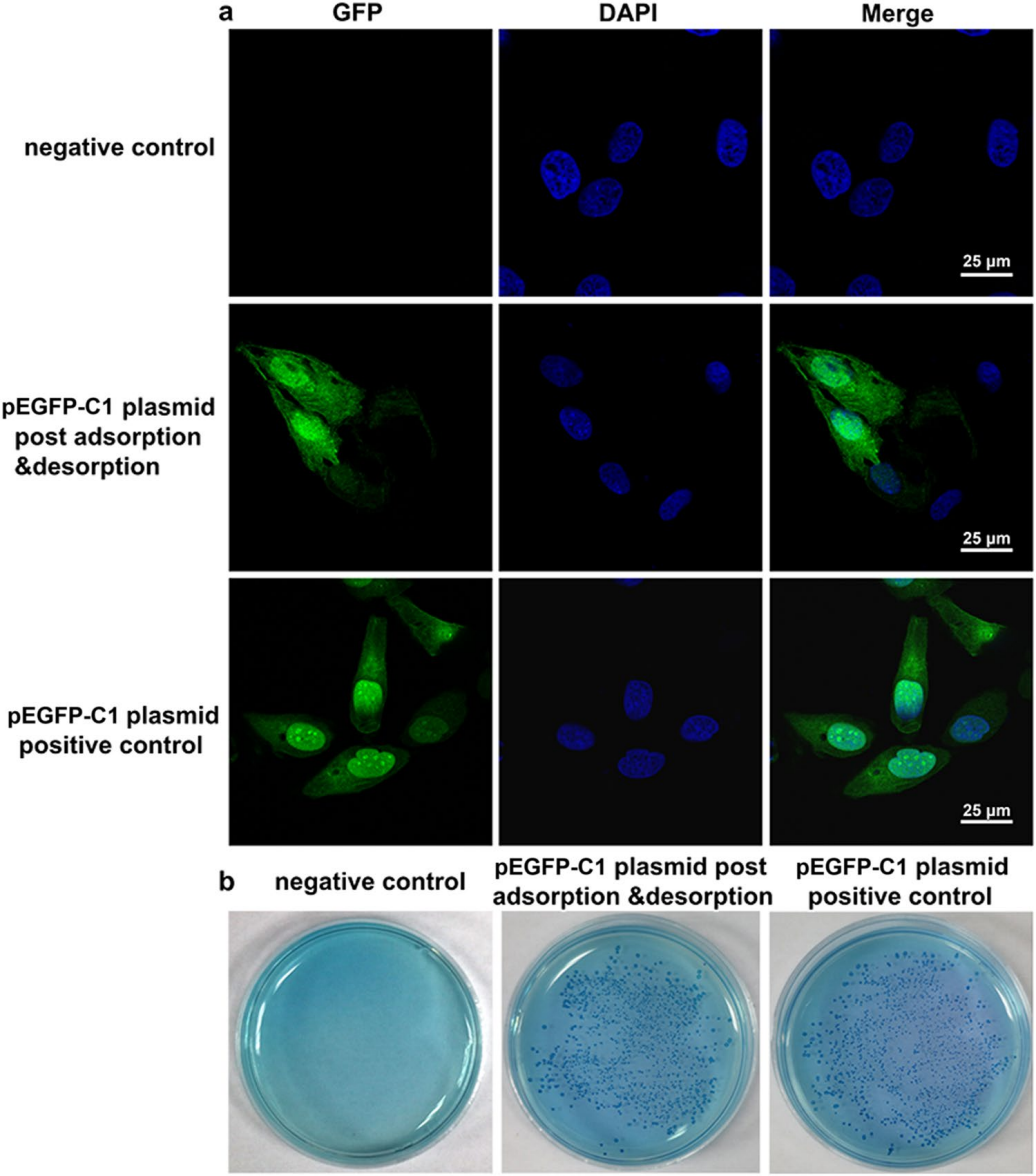
DNA eluted from ion-MMT do not effect the function of DNA (**a**) pEGFP-C1 plasmids maintained their expression function(green) after adsorption and desorption was transfected into HeLa. The nuclei was stained by DAPI (blue). (**b**) pEGFP-C1 plasmids maintained their expression function after adsorption and desorption was transformed to *E. coli*. The clones were counted after kanamycin selection. (**c**) Statistic analysis was performed for (**b**). ns, no significant difference.

Expression of the target gene in the host cells by plasmid transfection or transformation requires an integral plasmid DNA. Thus, the pEGFP-C1 plasmid expressing green fluorescent protein was chosen to validate the integrity of the eluted DNA. The pEGFP-C1 plasmid, which underwent an adsorption and desorption procedure, was transfected to the HeLa cells (Fig. 4a and Fig. S4). The eluted plasmid expressed GFP protein in HeLa cells, and green fluorescence was observed. In addition, similar results were obtained with the intact pEGFP-C1 plasmid (pEGFP-C1 plasmid positive control). No green fluorescence was observed in the HeLa cells without plasmid transfection (negative control). The same results were obtained when we transformed *E.coli* (Escherichia coli) with the eluted plasmid (Fig. 4b). These results confirmed that the eluted DNA is undamaged, functional and ready to perform multiple downstream applications.

## Discussion

Dispersion effects of ion-MMT modulate the adsorption efficiency. The classical DLVO theory is currently used as a benchmark to reasonably discuss the attractive or repulsive force between surfaces of charged particles, and further describe the dispersion and sedimentation of particles. Van der Waals attraction and electrostatic repulsion are involved into the stability of the particles’ solution. Essentially, the thickness of electrical double layer is a critical factor. The thickness of the electrical double layer is known as the Debye length κ^−1^ based on the following expression:

1 $$\kappa =\sqrt{(\frac{2{e}^{2}{N}_{A}\sum {Z}_{i}^{2}{C}_{i}}{\varepsilon kT})}$$

where e is the electronic charge (1.062 × 10^−19^ C), N_A_ is the constant of Avogadro 6.022 × 10^23^ mol^−1^, Z is the valency of the ion. Here, Li^+^ has a valency of +1, Ca^2+^ has a valency of +2 and Al^3+^ has a valence of +3, C is the molar concentration of ion, ε is the absolute dielectric constant of dispersive medium, k is the constant of Boltzmann 1.381 × 10^−23^ J, and T is the absolute temperature.

**Figure 5:**
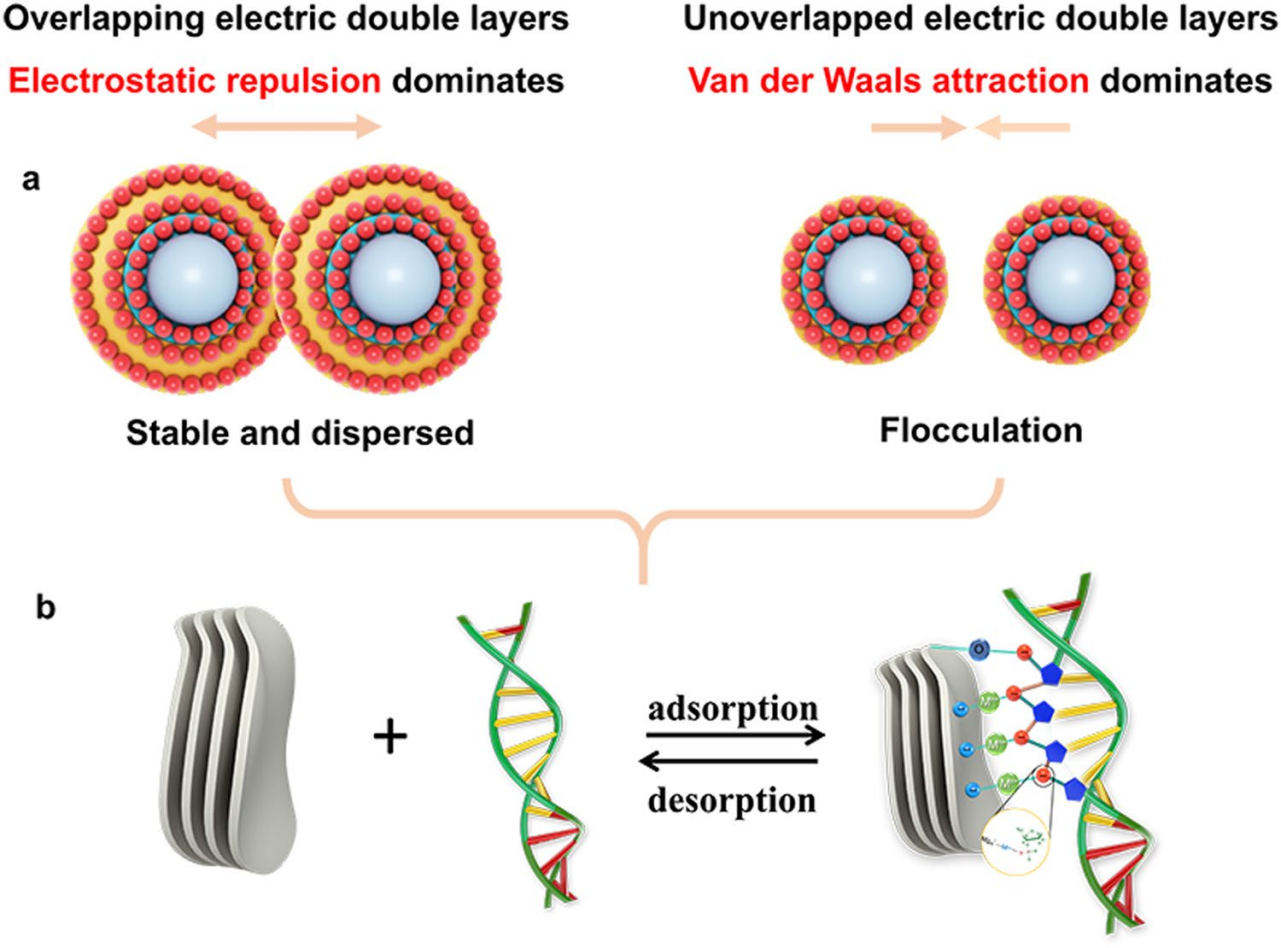
Surface charge and adsorption model of ion-MMT (**a**) Stabilization and flocculation model of dispersion system according to the DLVO theory. (**b**) Cation bridge of DNA adsorption by ion-MMT.

Apparently, the Debye length κ^−1^ is inversely proportional to the ion valence when C and ε remain the same in different samples. Suppose the charge is low, i.e., the Z value is small, the corresponding value of κ^−1^ is large, and the double electric layer formed is thicker. Two particles approach each other, and the interference between their electrical double layers increased and overlapped. Thus, the particle-particle electrostatic repulsion increases, and a relatively stable state was achieved. Simultaneously, when the electric double layer of the charged surface is thin, the Van der Waals attraction dominants, and the particles get closer. Thus, the particles’ solution tends to the sedimentation state (Fig. 5a).

As a result, the MMT solution’s stability depends on the different valence cations that are modified based on the electric double layer theory. Electric double layer thickness is reduced/increased with high/lower valence cations, and the minerals tended to be sedimentation- stable due to the Van der Waals attraction/electrostatic repulsion.

**Figure 6:**
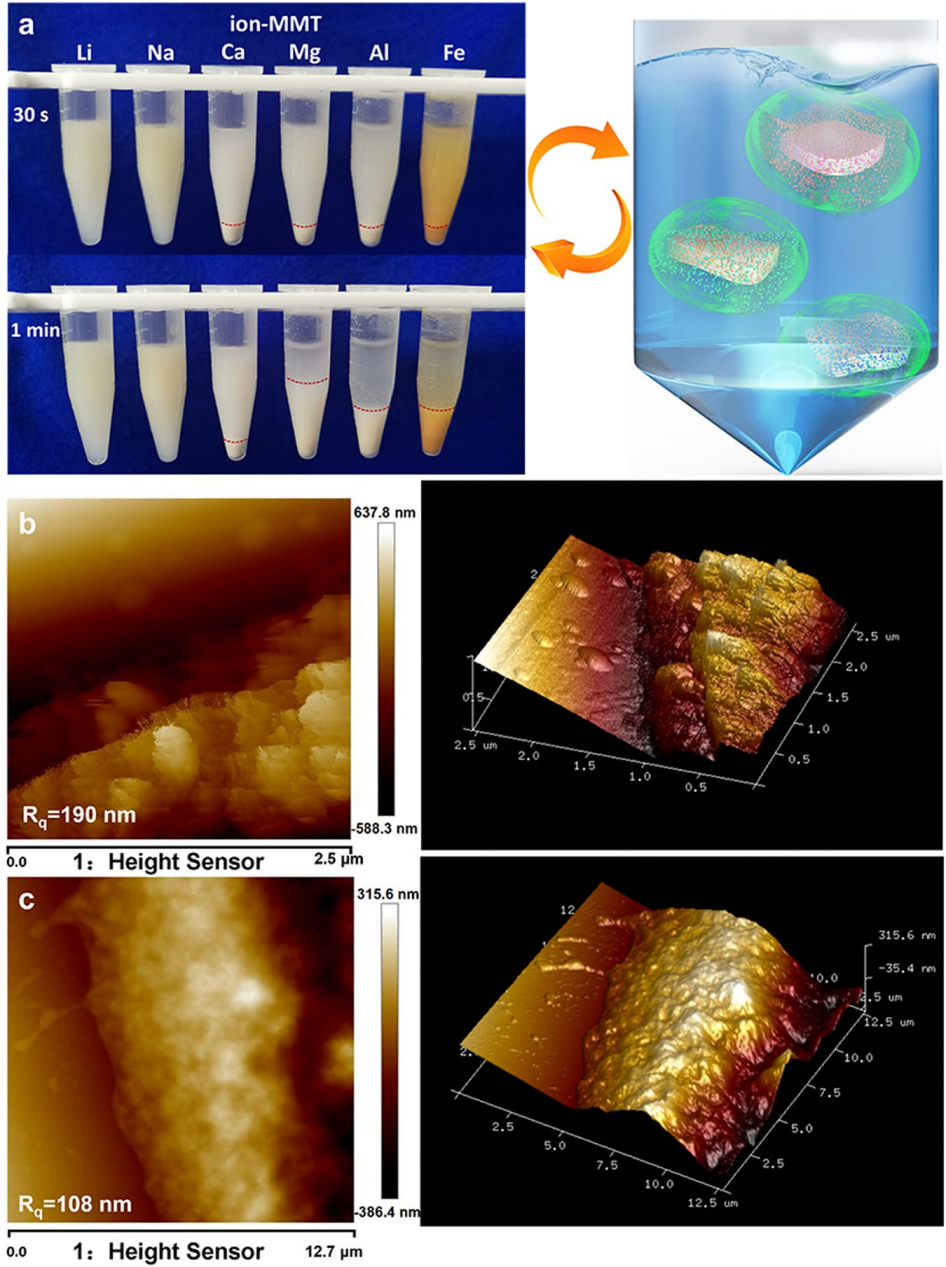
Sedimentation phenomenon and changes in surface morphology of MMT before and after DNA binding: (**a**) The dispersion of ion-MMT. Change of surface morphology: AFM topography image of Li-MMT before (**b**) and after (**c**) binding with DNA.

Further, the DNA adsorption was based on the cation bridge construction (Fig. 5b), but it was profoundly affected by the thickness of the electric double layer formation of the charged surface. The electric double layer thickness increased in the presence of lower valence cations (e.g., Li^+^, Na^+^). Minerals with lower charge states (more negative) tend to be stable due to the electrostatic repulsion (Fig. 6a); thus, the DNA could fully contact the MMT. The electric double layer thickness decreases in the presence of high valence cations (e.g., Fe^3+^, Al^3+^). Minerals with higher charge states (less negative) tend towards sedimentation due to the Van der Waals attraction (Fig. 6a); thus, only a few DNA are adsorbed on the MMT. Phenomenon of dispersion and agglomeration has an important bearing on the valence state of ions. The minimum concentration follows the Schulze-Hardy rule^42^:

2 $${\rm{l}}{\rm{o}}{\rm{g}}(ccc)\approx n\,{\rm{l}}{\rm{o}}{\rm{g}}(\frac{1}{Z})$$

where Z is the valency of the ion, and n is 6 in three dimensions. Therefore, the minimum concentration of monovalent, two valence and trivalent ions to trigger agglomeration is approximately 1: (1/2)^6^: (1/3)^6^. Metal cations with different valence of ion-MMT are consistent with this rule. Thus, rapid sedimentation can be achieved with low concentrations of high-valence metal cations. Generally, adsorption is difficult to occur between substances that are both negatively charged. Cations could act as a bridge and thus facilitate adsorption if it is introduced into the middle of particles. Repulsion forces will be generated between “bridges” with moderate length, which make particles simultaneously more disperse and stabile. Low-valence cations form “bridges” with moderate length (the double layer is thicker) on the surface of materials, the excellent dispersion make it possible for “bridges” to connect with DNA. The “bridge” formed by the high-valence cation on the surface of the material is shorter (the electric double layer is thinner), and the particles have been agglomerated before most of the “bridge” is attached to DNA, thereby reducing the amount of adsorption.

Most of the previous investigations believed that three adsorption modes exist: electrostatic interaction, cation bridge and ligand exchange. The electrostatic interaction is the dominant relaxation mechanism when the nanomaterial surface is positively charged. The cation bridge plays a major role when both the material surface and DNA are negatively charged. The ligand exchange is typically used to explain the interaction between the nanomaterial surface (with hydroxyl groups) and DNA (with the phosphate groups). In contrast to the existing literature reports, this paper proposed a creative DNA adsorption based on the electric double layer theory for material design and the cation bridge mechanism for interface analysis. In addition, the surface and interface analysis by the atom force microscope (AFM) illustrate that the roughness of the MMT surface was significantly reduced after DNA adsorption (Fig. 6b,c).

Natural two-dimensional (2D) MMT nanoclay has been incorporated into a DNA purification system. The interfacial interaction between ion-MMT and DNA was studied while maintaining the overall negative charge. The adsorption and desorption of DNA could be achieved by different ion-MMT combinations. The expression of green fluorescent protein from the eluted DNA indicates its excellent integrity. Based on the aforementioned results, we demonstrate a strong correlation between the DNA adsorption efficiency and the electric double layer construction. The DNA adsorption mechanism involves cation bridge construction and electric double layer formation which could be regulated by the surface charge. The degree of combination between DNA and ion-MMT was also strongly charge dependent. The lowest charge states (more negative) exhibit high adsorption behaviour with a maximum adsorption. Due to the electrostatic repulsion between particles with a thicker electric double layer, the lowest charge states make the system more dispersed and stable. As the charge state increases, the accelerated sedimentation of particles become stronger, due to the Van der Waals attraction of particles with thinner electric double layer. The electric double layer provides the desired cation for the cationic bridge to produce DNA adsorption. However, the dispersion and sedimentation of ion-MMT caused by the thickness of the electric double layer are more closely related to the adsorption efficiency. This study provides a detailed explanation for DNA adsorbing to MMT, which focuses on the in-depth analysis of the interfacial interaction during the non-destructive purification.

## Methods

### Materials

MMT obtained from Zhejiang, China was purified to produce ultrafine clay powders (>99% Ca-MMT). All chemicals were analytical-reagent grade. MgCl_2_·6H_2_O was purchased from Xilong Chemical Reagent Co., Ltd. The other chemicals, including Na_2_CO_3_, Li_2_CO_3_, and Fe(NO_3_)_3_·9H_2_O, Al(NO_3_)_3_·9H_2_O were purchased from Sinopharm Chemical Reagent Co., Ltd.

### Cationic modified montmorillonite

Briefly, 0.47 g of inorganic salt, including Na_2_CO_3_, Li_2_CO_3_, MgCl_2_·6H_2_O, Fe(NO_3_)_3_·9H_2_O, Al(NO_3_)_3_·9H_2_O and 1.00 g Ca-MMT, were respectively added to 9 mL of deionized water (including two aqueous solutions at pH 2 for trivalent ions), and the mixture suspension was mechanically stirred for 24 h at 60 °C. Among them, Ca-MMT was first added into the water prior to Li_2_CO_3_ for Li-MMT. Following this, the cationic modified MMT was precipitated by centrifugation. The final products were obtained after drying at 60 °C for 24 h.

### Characterization

Powder X-ray diffraction (XRD, RIGAKU D/max-2550 PC) measurements of the samples were performed with a DX-2700 X-ray diffractometer using Cu Kα radiation (λ = 0.15406 nm) at a scan rate of 0.02°/s. The Fourier transform infrared (FTIR) spectra of the samples were obtained using an IRAffinity-1 FTIR spectrophotometer with KBr pellets. Scanning electron microscopy (SEM) was performed using a TESCAN MIRA3 LMU scanning electron microscope at an accelerating voltage at 20 kV, which was equipped with an Oxford X-Max20 energy spectrum system and Gatan H1002 hot table system.

### Fragmented DNA preparation

Genomic DNA was purified from HeLa cells using phenol-chloroform extraction. Approximately 1.0 × 10^7^ HeLa cells were digested from 10 cm culture dish with 0.05% trypsin and washed twice in PBS. After washing, the cells were treated with proteinase K in 3 mL lysis buffer containing 2% SDS and 10 mM Tris-HCl, pH 8.0 overnight. The digestion mixture was extracted sequentially with 6 mL phenol and 6 mL chloroform. DNA was precipitated by adding the supernatant to 10 mL ethanol. The DNA precipitation was washed twice with 500 μL 70% ethanol and diluted in 1 mL double distilled water. The diluted DNA was then fragmented by 10 s sonication. Concentration of the fragmented DNA was detected using a spectrophotometer based on 260 nm absorbance. Then, the fragmented DNA was diluted to 200 ng/μL with double distilled water.

### Adsorption and desorption of DNA

Briefly, 30 mg of clay (Ca-MMT or modified MMT) was first added to 1 mL of double distilled water to make clay suspension. Then, 10 μL of MMT suspension (containing 300 μg of MMT) was then mixed with 50 μL fragmented DNA (containing 10 μg of DNA), 5 μL of 3 M NaAc (pH 5.0) and 35 μL double distilled water to make a 100 μL mixture. The mixture was gently shaken at RT for 5 min and centrifuged at 10,000 g for 10 min. DNA in the supernatant was spectrophotometrically accessed at 260 nm. The amount of DNA adsorbed was calculated by the difference between the amount of DNA added and that remaining in the supernatant.

The adsorption mixture described above was centrifuged at 10,000 g for 10 min. The residue was then washed with 100 μL double distilled water once followed by 100 μL TE buffer (containing 10 mM Tris, pH 8.0 and 1 mM EDTA) to elute the adsorbed DNA.

A similar adsorption and desorption procedure was performed with 10 μg pEGFP-C1 plasmid for further transfection experiments.

### Cell culture and transfection

HeLa cells were grown in DMEM/high glucose containing 10% FBS in 5% CO_2_. Before transfection, cells were seeded onto glass coverslips in 24-well plates, and plasmid transfection was performed with Lipofectamine 3000 reagent (Invitrogen, San Diego, USA) according to manufacturer’s protocol. Then, 500 ng pEGFP-C1 plasmid pre- or post-material adsorption and desorption were transfected to a unique well, and an equal quantity of intact pcDNA3.1 plasmid was used for negative control cells. After 48 hours, cells were fixed for immunofluorescence.

### Immunofluorescence

Forty-eight hours after transfection, HeLa cells were washed twice with PBS, fixed with paraformaldehyde (Sigma-Aldrich, St. Louis, USA) (4% w/v) for 15 min, and permeabilized with 0.1% PBST (Triton X-100 in PBS) for 5 min. The coverslips were then incubated with 4′,6-diamidino-2-phenylindole(DAPI; Sigma-Aldrich, St.Louis, USA) for 5 min to stain cell nuclei. The cells were then mounted with ProLong Gold Antifade mountant (Thermo Fisher Scientific, Waltham, USA), and observed using laser-scanning confocal microscope (TCS SP5, Leica, Wetzlar, Germany).

## Supplementary information

Supplementary Information
