# Supplementary material for: Charge-Dependent Regulation in DNA Adsorption on 2D Clay Minerals
Source: Sci Rep. 2019 May 2;9:6808. doi: 10.1038/s41598-019-41093-5 (PMC6497631; doi:10.1038/s41598-019-41093-5)
Supplement: Supplementary file 1 — Supplementary Information [file 41598_2019_41093_MOESM1_ESM.doc]

**Electronic Supplementary Information**

Charge-Dependent Regulation in DNA Adsorption on 2D Clay Minerials

Hongyi Xie, ^†, ‡, #^ Zhengqing Wan, ^§, #^ Song Liu, ^⊥^ Yi Zhang, ^†, ‡, *^ Jieqiong Tan, ^§, *^ and Huaming Yang ^†, ‡, *^

^†^ Department of Inorganic Materials, School of Minerals Processing and Bioengineering, Central South University, Changsha 410083, China

^‡^ Hunan Key Lab of Mineral Materials and Application, Central South University, Changsha 410083, China

^§^ The Center for Medical Genetics, School of Life Science, Central South University, Changsha 410078, China

^⊥^ Institute of Chemical Biology and Nanomedicine, College of Chemistry and Chemical Engineering, Hunan University, Changsha 410082, China.

* E-mail: yee_z10@csu.edu.cn (Y. Zhang); tanjieqiong@sklmg.edu.cn (J. Tan); hmyang@csu.edu.cn (H. Yang)

# These authors contributed equally.

**Figures captions**

**Table S1.** Summary of adsorption pattern of different materials

**Figure S1.** The adsorption and desorption of DNA by ion-MMT in different adsorption time.

**Figure S2.** XRD pattern of Na-MMT, Li-MMT, Ca-MMT, Mg-MMT, Fe-MMT and Al-MMT

**Figure S3.** FTIR spectra of ion-MMT before and after binding with DNA

**Figure S4.** Representative fluorescence microscopy images of transduced cells at 48 h post-transfection.

**Table S1**. Summary of adsorption pattern of different materials

| Materials | Adsorption pattern | Ref. |
| --- | --- | --- |
| mesoporous silica nanoparticles | direct and indirect hydrogen bonds | S1 |
| aminosilane-modified magnetic nanoparticles | electrostatic interactions (amino groups) | S2 |
| magnetic nanoparticles | affinity ligand | S3 |
| indium tin oxide nanoparticles | electrostatically adsorb (surface charge) | S4 |
| Graphene oxide | π-π stacking and hydrogen bonding | S5 |
| MoS_2_ and WS_2_ | Van der Waals force | S5 |
| present work | cation bridge and electric double layer |  |


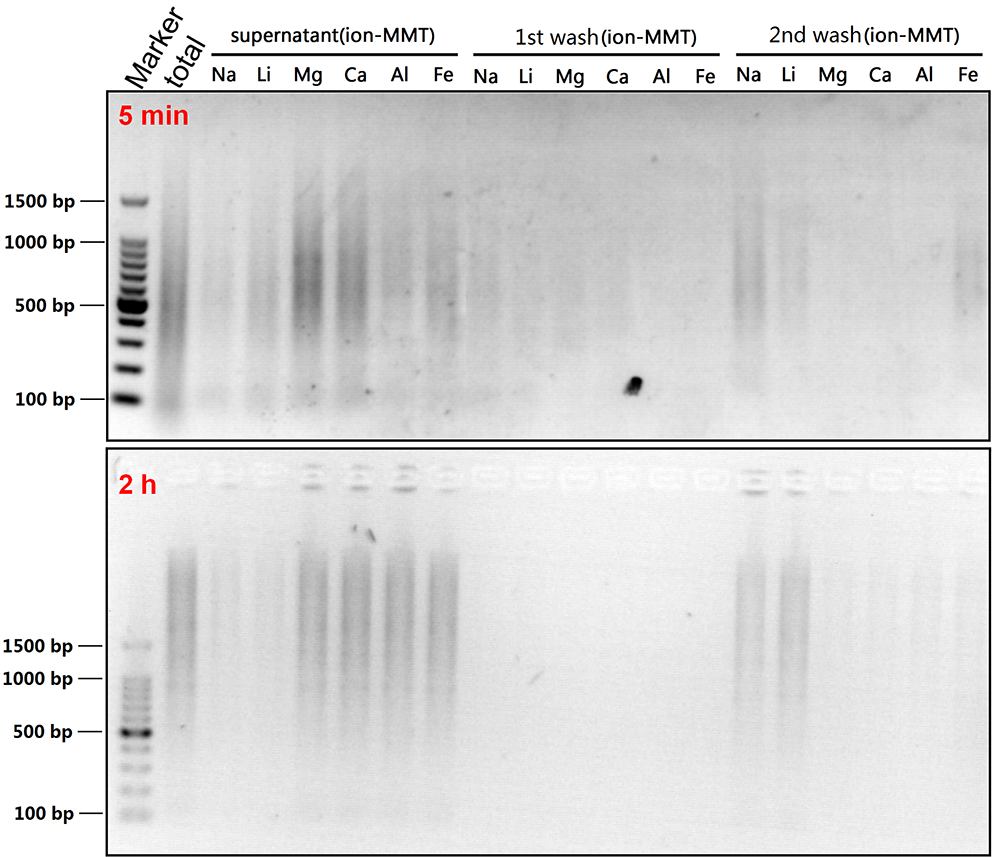


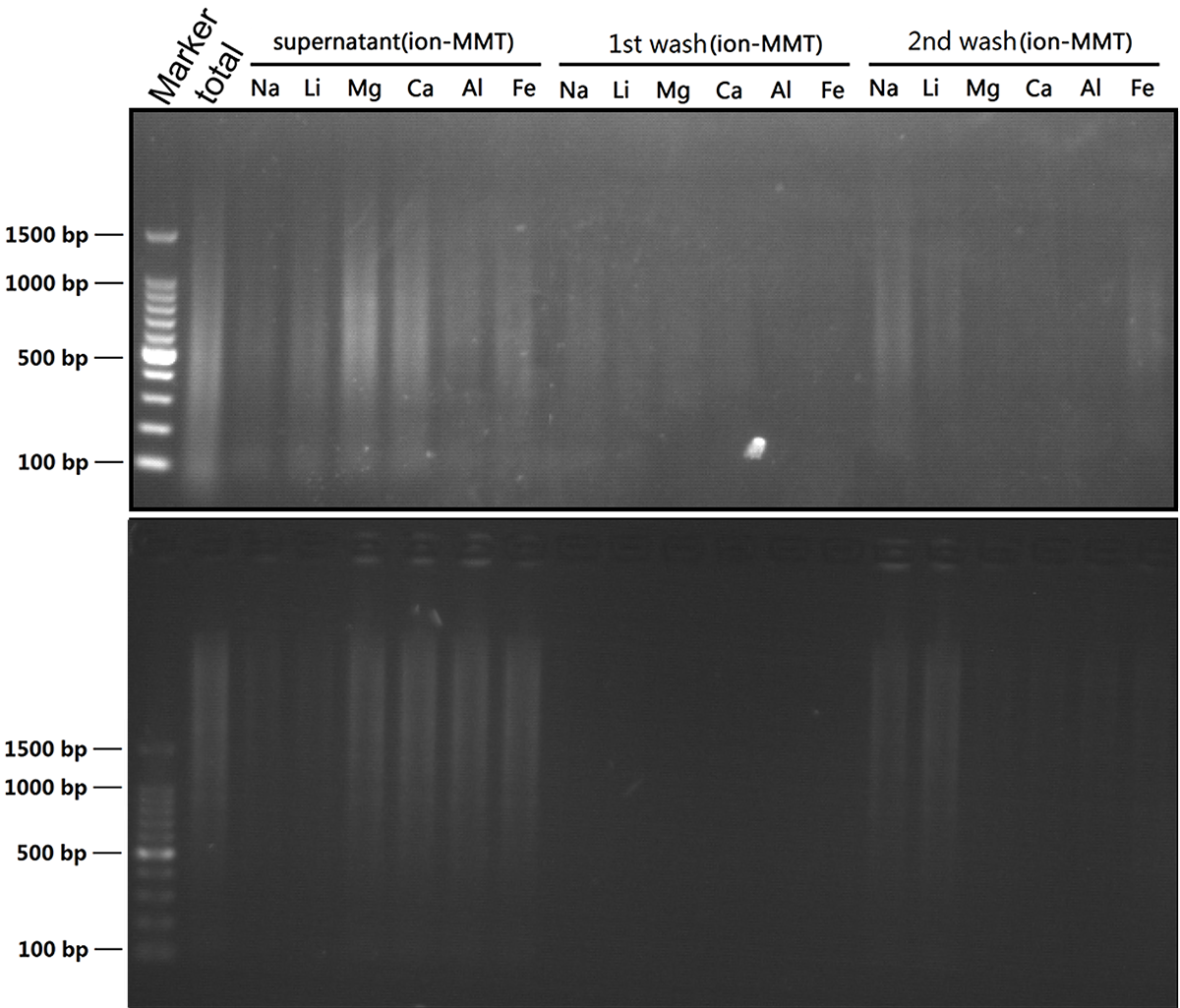


**Figure S1**. The adsorption and desorption of DNA by ion-MMT in different adsorption time with multiple exposures. A better performance arose in Li-MMT and Na-MMT, which followed by Al-MMT and Fe-MMT when the adsorption is 5 min. The adsorption of Mg-MMT and Ca-MMT is relatively poor. The adsorption of Al-MMT and Fe-MMT became weaker when the adsorption time is extended to 2 hours. For the desorption process, negligible DNA desorption in the first wash, whereas a significant DNA desorption occurred during the second wash.


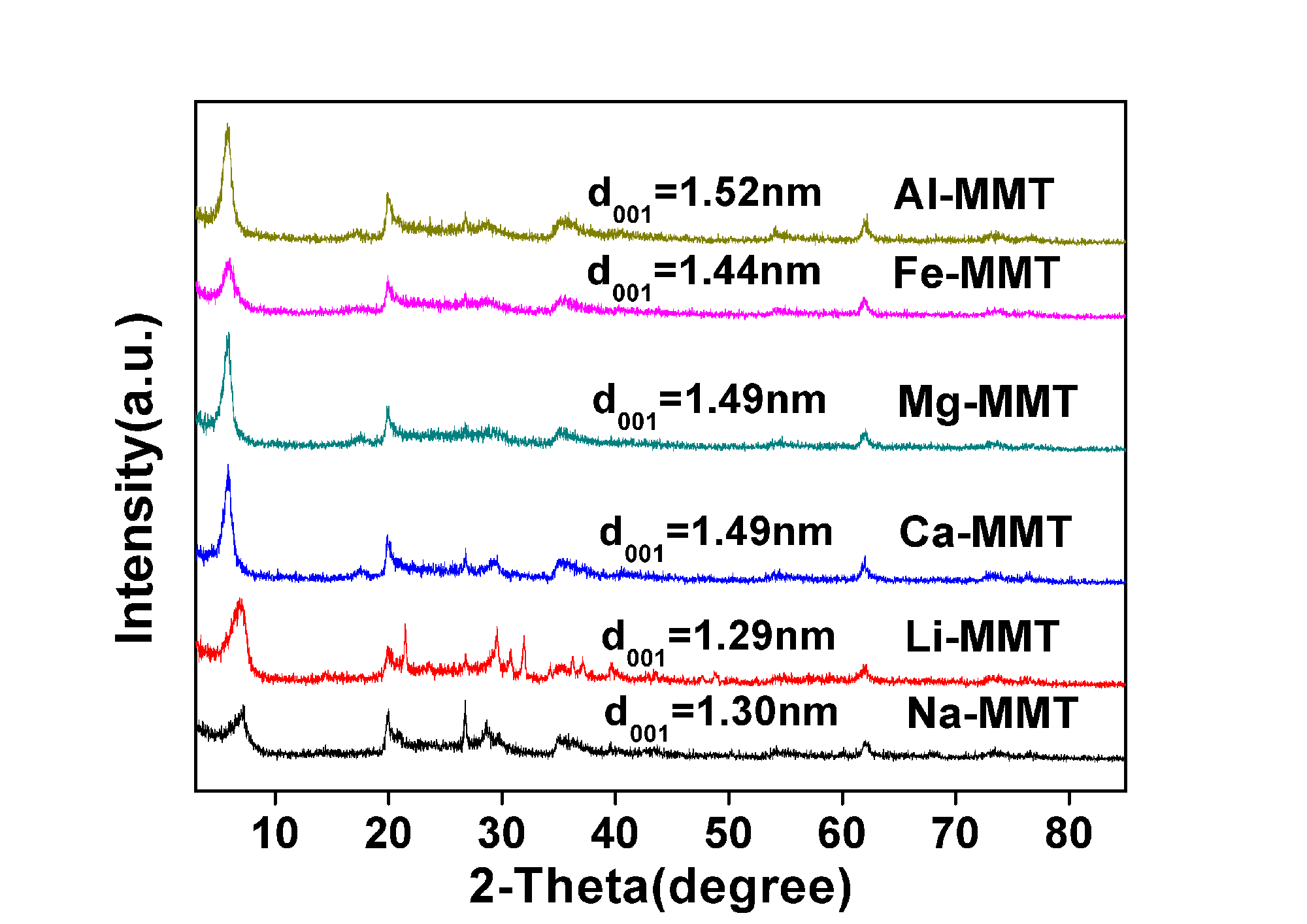


**Figure S2**. XRD pattern of Na-MMT, Li-MMT, Ca-MMT, Mg-MMT, Fe-MMT and Al-MMT


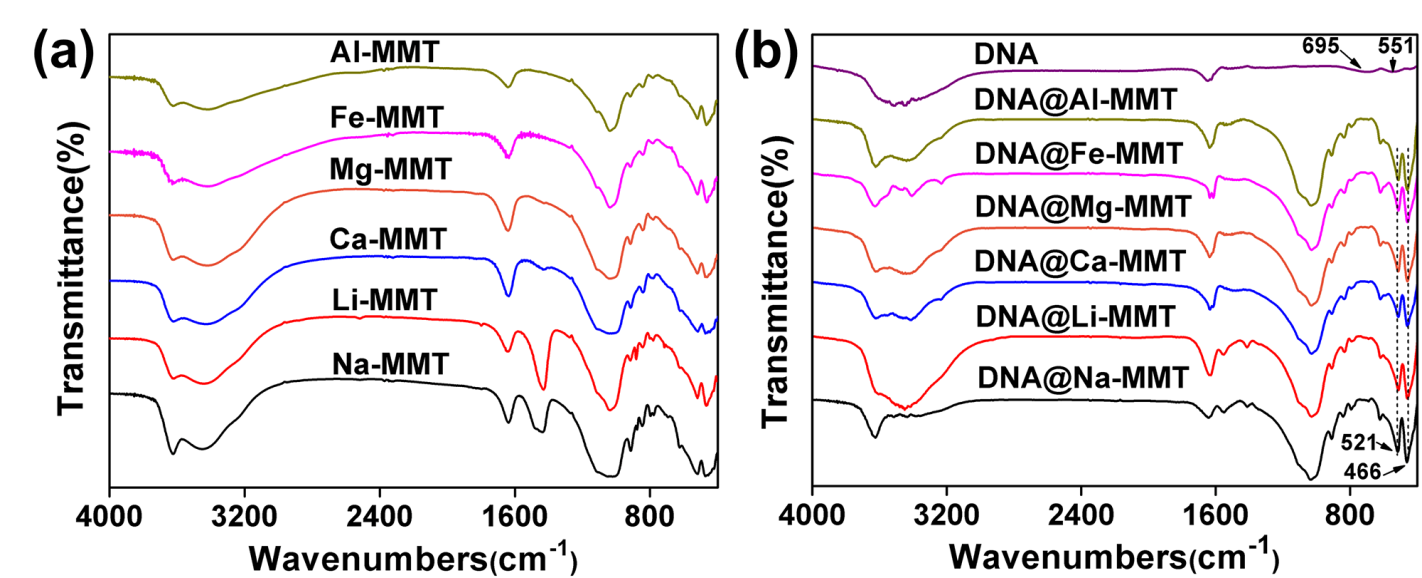


**Figure S3**. FTIR spectra of ion-MMT before (a) and after (b) binding with DNA


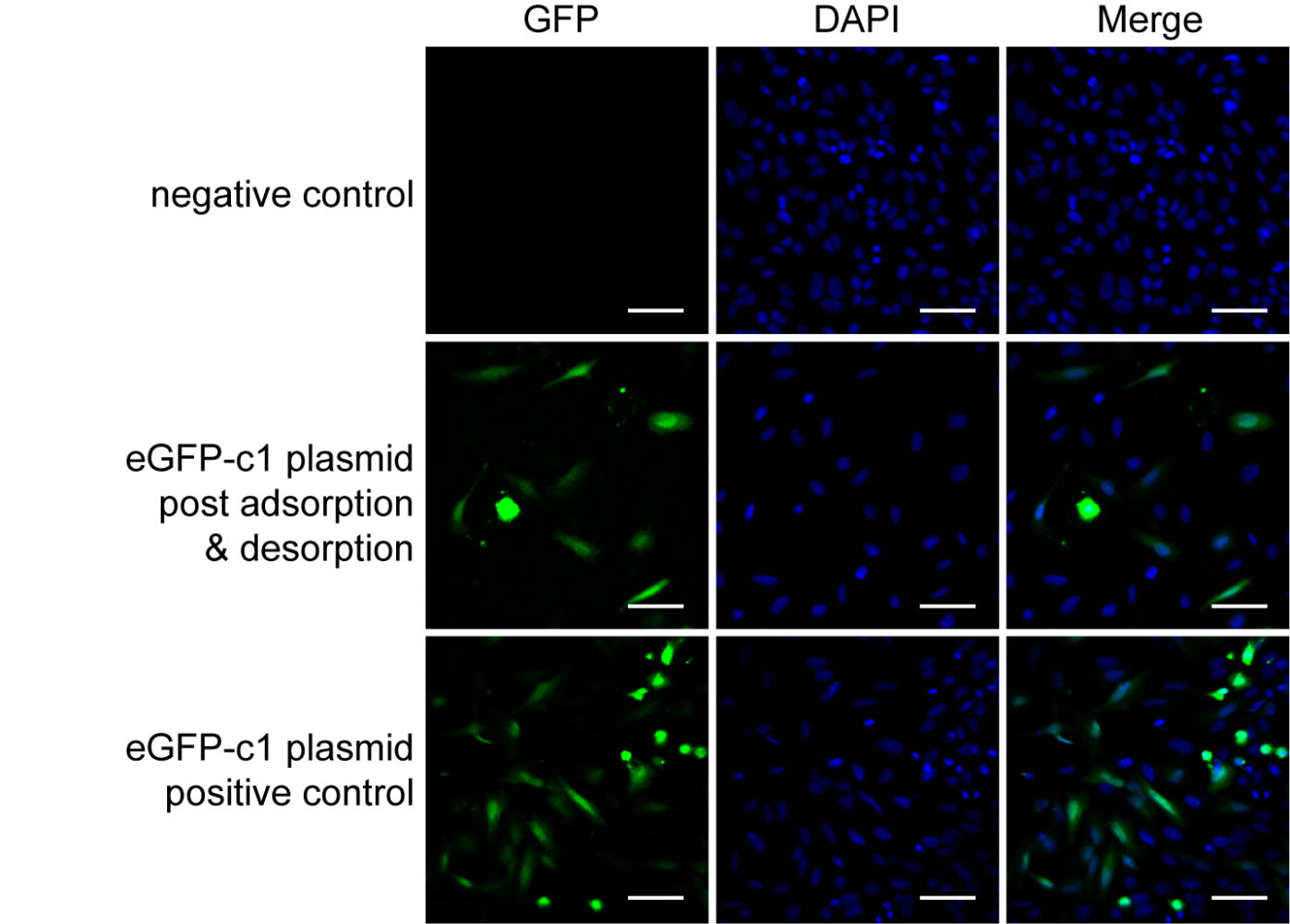


**Figure S4**. Representative fluorescence microscopy images of transduced cells at 48 h post-transfection. Scale bar: 75 μm.

**Supplementary References:**

1. Li X, Zhang J, Gu H. Study on the adsorption mechanism of DNA with mesoporous silica nanoparticles in aqueous solution. Langmuir 2012; 28: 2827-2834.
2. Tanaka T, Sakai R, Kobayashi R, et al. Contributions of phosphate to DNA adsorption/desorption behaviors on aminosilane-modified magnetic nanoparticles. Langmuir 2009; 25: 2956-2961.
3. Probst CE, Zrazhevskiy P, Gao X. Rapid multitarget immunomagnetic separation through programmable DNA linker displacement. J Am Chem Soc 2011; 133: 17126-17129.
4. Liu B, Liu J, DNA adsorption by indium tin oxide nanoparticles. Langmuir 2015; 31: 371-377.
5. Lu C, Liu Y, Ying Y, et al. Comparison of MoS_2_, WS_2_, and graphene oxide for DNA adsorption and sensing. Langmuir 2017; 33: 630-637.
